# Spleen stiffness measurements using point shear wave elastography detects noncirrhotic portal hypertension in human immunodeficiency virus

**DOI:** 10.1097/MD.0000000000017961

**Published:** 2019-11-22

**Authors:** Ayesha K. Ahmad, Sebastiana Atzori, Simon D. Taylor-Robinson, James B. Maurice, Graham S. Cooke, Lucy Garvey

**Affiliations:** a Department of Surgery and Cancer; b Division of Medicine; c Department of Infectious Diseases, Imperial College, St Mary's Hospital Campus, London W2 1PG; d Jefferiss Wing, Department of HIV Medicine, Imperial College Healthcare NHS Trust, St Mary's Hospital, London W2 1NY.

**Keywords:** human immunodeficiency virus, liver stiffness, non-cirrhotic portal hypertension, point shearwave elastography, spleen stiffness

## Abstract

To assess the utility of spleen stiffness as a diagnostic tool in individuals with human immunodeficiency virus (HIV) and non-cirrhotic portal hypertension (NCPH).

The Philips EPIQ7, a new point shearwave elastography (pSWE) technique, was used to assess liver and spleen stiffness in 3 patient groups. Group 1: HIV and NCPH (n = 11); Group 2: HIV with past didanosine (ddI) exposure without known liver disease or NCPH (n = 5); Group 3: HIV without known liver disease or ddI exposure (n = 9).

Groups were matched for age, HIV chronicity, and antiretroviral treatment (including cumulative ddI exposure in Groups 1 and 2). Differences in liver and spleen stiffness (in kPa) between groups were analyzed using the Mann–Whiney *U* test.

Liver and spleen stiffness were both significantly higher in NCPH versus ddI-exposed (*P* = .019 and *P* = .006) and ddI-unexposed controls (*P* = .038 and *P* < .001). Spleen stiffness was more effective than liver stiffness at predicting NCPH, area under receiver operating characteristic (AUROC) 0.812 versus 0.948. Combining the 2 variables improved the diagnostic performance, AUROC 0.961. The optimal cut-off for predicting NCPH using splenic stiffness was 25.4 kPa, with sensitivity 91%, specificity 93%, positive predictive value (PPV) 91%, negative predictive value (NPV) 93%, positive likelihood ratio 12.73, negative likelihood ratio 0.10. Spleen and liver stiffness scores were strongly correlated (*P* = .0004, 95% confidence interval [CI] 18, 59).

Elevated spleen stiffness is observed in HIV with NCPH and can be quantified easily using pSWE with high diagnostic accuracy. Novel strategies such as pSWE for longitudinal monitoring of patients with HIV and NCPH should be considered.

## 1 Introduction

Portal hypertension in the absence of overt parenchymal chronic liver disease has been described in small cohorts of patients with human immunodeficiency virus (HIV) with complications including variceal bleeding and ascites.^[1–3]^ Liver biopsy in these patients has commonly revealed features of nodular regenerative hyperplasia without liver cirrhosis, resulting in the development of non-cirrhotic portal hypertension (NCPH) with varices and splenomegaly. Liver histology and portal venous pressure measurements remain the gold standard for definitive diagnosis,^[4]^ but are limited by their invasive and expensive nature, and therefore cannot be used for longitudinal monitoring and follow-up. These findings have primarily been attributed to didanosine (ddI) exposure, leading to the Federal Drug Agency (FDA) warning of NCPH and its use in clinical practice has now declined.^[5]^ While this may avert further new diagnoses of HIV NCPH, the optimal management of established HIV NCPH patients is yet to be standardized.

Ultrasound elastography is now increasingly used as a non-invasive tool to identify hepatic fibrosis in a wide range of liver conditions with high positive and negative predictive values.^[6,7]^ Transient elastography (TE), the most validated ultrasound elastography technique, has recently been shown to have an association with the severity of portal hypertension in those with NCPH of various etiologies.^[8]^ However, given the wide range of liver stiffness measurements (LSM) that are described in NCPH, the utility of LSM using TE remains limited.^[8]^

Point shearwave elastography (pSWE) is a novel form of elastography assessment, which has demonstrated similar sensitivity and specificity as TE in detecting fibrosis in individuals,^[9,10]^ but it overcomes the main limitations of TE, namely its applicability in patients with large body habitus or ascites, simultaneous ultrasound morphological and fibrosis assessment and also an ability to assess more than one organ during the same examination without requiring recalibration.^[9,10]^

The spleen has now also become an organ of interest in the identification of portal hypertension and associated extra-hepatic complications such as esophageal varices.^[11,12]^ Spleen stiffness measurements (SSM) have shown a superior association with the presence of portal hypertension compared with LSM in cirrhosis patients.^[12,13]^ Thus far, neither splenic assessment or use of pSWE has been utilized in HIV cohorts as a potential indirect non-invasive marker of liver and portal system disease.

In this study, we aimed to assess SSM and LSM, measured by pSWE, in patients with and without NCPH, the latter stratified for prior ddI exposure.

## 2 Methods

### 2.1 Study design

In this prospective cross-sectional study, patients were recruited at the HIV Outpatient Department, Imperial College Healthcare NHS Trust. All patients were adults (over 18 years) and were receiving treatment for chronic HIV-1 antibody infection. It was a requirement of the study entry that patients had undetectable (<20 c/mL) plasma HIV RNA level for a minimum of 6 months. Patients were further stratified into 3 groups with the following inclusion criteria: Group 1: HIV + NCPH (defined as the presence of portal hypertension manifestations in the absence of cirrhosis diagnosed by liver biopsy); Group 2: HIV + past ddI exposure (without known NCPH or liver disease); Group 3: HIV and no ddI exposure or history of liver disease. Exclusion criteria included active infective or opportunistic illness, malignancy, pregnancy, cardiac failure, current hepatitis B/C infection, significant liver disease due to any cause and alcohol consumption in excess of current UK recommended limits. Groups were matched for age, HIV chronicity, and antiretroviral treatment (including cumulative ddI exposure in Groups 1 and 2). pSWE was performed using Philips EPIQ7 (ElastPQ, Philips Medical Systems, Seattle, WA). Patients were placed supine with arms abducted from the ultrasound probes and 10 stiffness measurements were taken from the right lobe of the liver and from the spleen. The median stiffness and IQR values were calculated for each region.

### 2.2 Ethics statement

This study was performed in accordance with the 1975 Declaration of Helsinki, with approvals from the Research Ethics Committee and the Joint Research and Compliance Office of Imperial College London and Imperial College Healthcare NHS Trust. Written informed consent was obtained from all patients (REC number: 15/LO/1749).

### 2.3 Statistics

Clinical and demographic data were collected. Associations between these parameters and liver and spleen stiffness were investigated using univariate linear regression analysis. Differences in liver and spleen stiffness (in kPa) between groups were analyzed using the Mann–Whiney *U* test. Diagnostic accuracy of spleen and liver stiffness in predicting the presence of NCPH was conducted using receiver operating characteristic (ROC) curves. Comparison of ROC curves using DeLong et al^[14]^ methodology was used to compare differences between diagnostic tests.

## 3 Results

### 3.1 Study population

A total of 25 patients were recruited and included in the final analysis: 11 patients with HIV and NCPH (group 1), 5 with HIV and ddI exposure, but no NCPH (group 2), and 9 with HIV and no ddI exposure or NCPH (group 3). Clinical characteristics of patients are summarized in Table 1. The mean age was 49.7 years, 56% were men and 21 (84%) patients had an undetectable HIV RNA level. Current mean CD4+ count was 497 cells/μL and mean Nadir CD4+ count was 162 cells/μL. The mean time since HIV diagnosis was 19 years. ddI exposed patients had a cumulative mean of 54 months exposure.

**Table 1:**
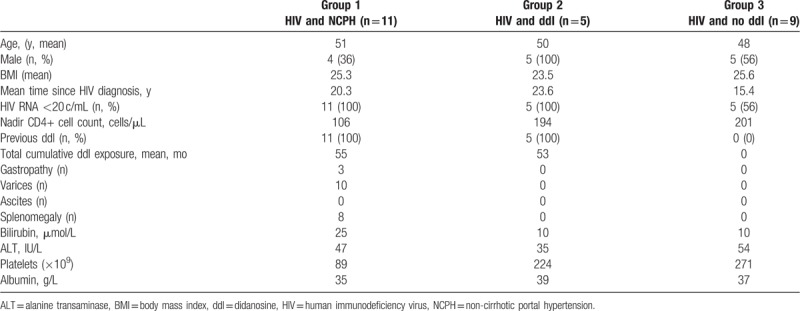
Baseline characteristics of the patient population.

### 3.2 Liver and spleen stiffness

**Figure 1:**
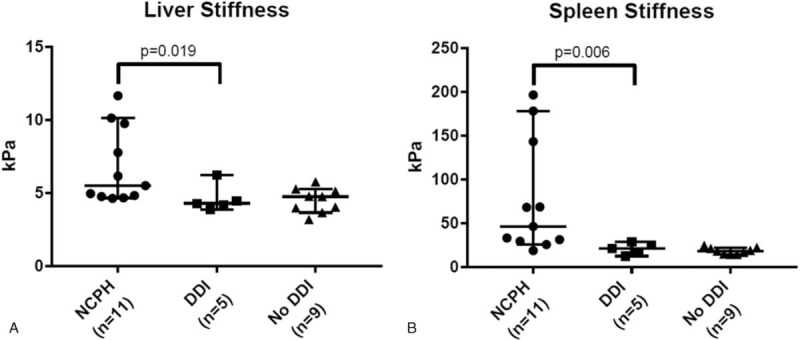
Horizontal axis: study group. Vertical axis: stiffness score in kilopascals (kPa). A and B: Liver and spleen stiffness scores (in kPa) using point shearwave elastography in 3 study groups.

Liver and spleen stiffness were both significantly higher in HIV patients with NCPH compared with ddI-exposed HIV patients and ddI-unexposed HIV controls (Fig. 1A and B). The median liver stiffness score in the NCPH cohort was 6.8 kPa (IQR: 2.53 kPa, range: 5–12 kPa), compared with 4.6 kPa (IQR: 1.27 kPa, range: 4–6 kPa, *P* = .019) in ddI-exposed and 4.5 kPa (IQR: 1.03 kPa, range: 3–6 kPa, *P* = .038) in ddI-unexposed individuals. Similarly, median spleen stiffness scores were higher in the NCPH cohort (76.3 kPa, IQR: 41.76 kPa, range: 18–197 kPa) than in the ddI-exposed (20.82 kPa, IQR: 5.52 kPa, range: 16.6–28.7 kPa, *P* = .006) and ddI-unexposed groups (18.36 kPa, IQR: 6.63 kPa, range: 14.1–24.6 kPa, *P* ≤ .001). Spleen and liver stiffness scores were strongly correlated (*P* = .0004).

Overall, higher spleen stiffness was significantly associated with lower nadir CD4+ cell count (*P* < .0001, 95% CI -561, -344), lower current CD4+ cell count (*P* < .0001, 95% CI -167, -69), lower platelet count (*P* < .0001, 95% CI -184, -90), and higher serum bilirubin (*P* = .01, 95% CI 6.3, 49). However, no significant associations were found between serum ALT, albumin or cumulative ddI exposure (*P* > .05 for all measures).

Diagnostic performance through measures of area under receiver operating characteristic (AUROC) curves demonstrated that spleen stiffness showed non-statistically significant improvement compared with liver stiffness at predicting NCPH (AUROC 0.948 vs 0.812, *P* = .1118). When combining the 2 variables, diagnostic accuracy improved, however this was statistically insignificant (AUROC 0.961, *P* = .2949). The optimal cut-off for predicting NCPH using splenic stiffness was 25.4 kPa, with sensitivity of 91%, specificity of 93%, positive predictive value of 91%, and negative predictive value of 93% (positive likelihood ratio 12.73, negative likelihood ratio 0.10).

## 4 Discussion

Currently, there is no consensus on the optimal long-term management strategy for the small numbers of people living with HIV and NCPH in the UK.^[15]^ We have demonstrated a potential non-invasive technique that can be used in the diagnosis and follow-up of such patients.

The sequelae of portal hypertension, including splenomegaly, portal venous congestion, and tissue hyperplasia may contribute to increased splenic stiffness, and therefore be associated with NCPH even in the absence of hepatic parenchymal damage.^[16]^ Non-invasive splenic stiffness assessment is presently being evaluated and shows promising use in portal hypertension caused by advanced chronic liver disease.^[17]^ Several techniques by which splenic stiffness can be assessed have been identified, but only TE has been previously used in NCPH.^[8,18]^ However, these studies have only assessed the use of liver stiffness and both present conflicting conclusions on its use as a potential diagnostic tool in NCPH of various etiologies.

To the best of our knowledge, this is the first paper to evaluate the use of spleen stiffness and pSWE to determine its utility in HIV patients with NCPH. We observed elevated spleen stiffness in our NCPH cohort, compared with ddI-exposed and unexposed controls. Moreover, our data suggest that NCPH can be quantified easily using pSWE with high diagnostic accuracy. Although further research is needed to validate our findings, the superior diagnostic value of pSWE compared with others, such as TE, is well established in cirrhotic portal hypertension.^[19–21]^ The mechanism by which some ddI-exposed individuals progress to NCPH, while others have no disruption to portal pressure, remains poorly understood.

Clearly our study is limited in its small and preliminary nature. While a larger sized study is desirable, the rare nature of this condition (HIV and NCPH) limits greatly the number of case subjects at our center. Nevertheless, we endeavored to match groups carefully according to clinical parameters (e.g., age, sex, HIV chronicity, and ddI exposure). We acknowledge that while we have demonstrated significant differences in spleen stiffness between groups, results should be interpreted with caution given the small size of the cohorts.

In summary, there are several clinical implications that arise from this study that are worthy of consideration. pSWE provides a rapid, non-invasive tool carrying minimal complications with higher applicability over other non-invasive techniques. It can also be implemented on regular ultrasound machines, which is likely to be both time-efficient and cost-effective for healthcare providers. As a result, novel strategies such as pSWE should be considered for longitudinal monitoring of HIV-positive individuals with NCPH. Future studies are needed to corroborate our findings, but we believe that spleen stiffness using ultrasonography is likely to have a significant positive impact in this small cohort of patients and further correlation of spleen stiffness and clinical outcomes should be evaluated.

## Acknowledgments

Philips Medical Systems (Seattle, WA) provided the Philips EPIQ7^TM^ system on loan to Imperial College London for this investigator-led study. The authors thank Mr James Jago and his team at Philips Medical Systems for their technical assistance with instrument set-up. They would like to thank all patients that participated in this work.

## Author contributions

**Conceptualization:** Simon D. Taylor-Robinson, Graham S. Cooke, Lucy Garvey.

**Data curation:** Lucy Garvey.

**Formal analysis:** Sebastiana Maria Atzori, James B. Maurice, Lucy Garvey.

**Investigation:** Sebastiana Maria Atzori, Lucy Garvey.

**Methodology:** Simon D. Taylor-Robinson, Graham S. Cooke, Lucy Garvey.

**Project administration:** Lucy Garvey.

**Resources:** Simon D. Taylor-Robinson.

**Software:** Lucy Garvey.

**Visualization:** Simon D. Taylor-Robinson.

**Writing – original draft:** Ayesha Karim Ahmad, Lucy Garvey.

**Writing – review & editing:** Simon D. Taylor-Robinson, James B. Maurice, Graham S. Cooke, Lucy Garvey.

Ayesha Karim Ahmad orcid: 0000-0003-3763-1212.
